# Supplementary material for: Sustained treatment of retinal vascular diseases with self-aggregating sunitinib microparticles
Source: Nat Commun. 2020 Feb 4;11:694. doi: 10.1038/s41467-020-14340-x (PMC7000758; doi:10.1038/s41467-020-14340-x)
Supplement: Supplementary file 2 — Description of Additional Supplementary Files [file 41467_2020_14340_MOESM2_ESM.pdf]

Description of Additional Supplementary Files

File name: Supplementary Movie 1

Description: Aggregate of sunitinib MPs in rabbit vitreous one day after intravitreous injection of MPs.
