# Supplementary material for: Sustained treatment of retinal vascular diseases with self-aggregating sunitinib microparticles
Source: Nat Commun. 2020 Feb 4;11:694. doi: 10.1038/s41467-020-14340-x (PMC7000758; doi:10.1038/s41467-020-14340-x)
Supplement: Supplementary file 4 — Reporting Summary [file 41467_2020_14340_MOESM4_ESM.pdf]

nature research

Corresponding author(s): Ming Yang, Justin Hanes, Peter Campochiaro

Last updated by author(s): Dec 9, 2019

Reporting Summary

Nature Research wishes to improve the reproducibility of the work that we publish. This form provides structure for consistency and transparency in reporting. For further information on Nature Research policies, see Authors & Referees and the Editorial Policy Checklist .

Statistics

For all statistical analyses, confirm that the following items are present in the figure legend, table legend, main text, or Methods section.

n/a	Confirmed	
☐	☒	The exact sample size (*n*) for each experimental group/condition, given as a discrete number and unit of measurement
☐	☒	A statement on whether measurements were taken from distinct samples or whether the same sample was measured repeatedly
☐	☒	The statistical test(s) used AND whether they are one- or two-sided<br>*Only common tests should be described solely by name; describe more complex techniques in the Methods section.*
☐	☒	A description of all covariates tested
☐	☒	A description of any assumptions or corrections, such as tests of normality and adjustment for multiple comparisons
☐	☒	A full description of the statistical parameters including central tendency (e.g. means) or other basic estimates (e.g. regression coefficient) AND variation (e.g. standard deviation) or associated estimates of uncertainty (e.g. confidence intervals)
☒	☐	For null hypothesis testing, the test statistic (e.g. *F*, *t*, *r*) with confidence intervals, effect sizes, degrees of freedom and *P* value noted<br>*Give P values as exact values whenever suitable.*
☒	☐	For Bayesian analysis, information on the choice of priors and Markov chain Monte Carlo settings
☒	☐	For hierarchical and complex designs, identification of the appropriate level for tests and full reporting of outcomes
☒	☐	Estimates of effect sizes (e.g. Cohen's *d*, Pearson's *r*), indicating how they were calculated

Our web collection on statistics for biologists contains articles on many of the points above.

Software and code

Policy information about availability of computer code

Data collection	1) Fluorescein angiography- Micron III Retinal Imaging Microscope(Phoenix Research Laboratories<br>2) ELISA-SpectraMax Plus (Molecular Devices).<br>3) Imaging-Zeiss Axioskop 2 Plus microscope (Zeiss)<br>4)Western blotting-ChemiDoc XRS (BioRad)
Data analysis	1) CNV image analysis- Image-Pro Plus (Media Cybernetics)<br>2) Western blotting densitometry-Image Lab software (BioRad)<br>3) Statistical Analysis- GraphPad Prism 6 (GraphPad Software)

For manuscripts utilizing custom algorithms or software that are central to the research but not yet described in published literature, software must be made available to editors/reviewers. We strongly encourage code deposition in a community repository (e.g. GitHub). See the Nature Research guidelines for submitting code & software for further information.

Data

Policy information about availability of data

All manuscripts must include a data availability statement. This statement should provide the following information, where applicable:

- Accession codes, unique identifiers, or web links for publicly available datasets
- A list of figures that have associated raw data
- A description of any restrictions on data availability

All data generated during this study are included in this published article (and its supplementary information files).

Field-specific reporting

Please select the one below that is the best fit for your research. If you are not sure, read the appropriate sections before making your selection.

- [x] Life sciences
- [ ] Behavioural & social sciences
- [ ] Ecological, evolutionary & environmental sciences

For a reference copy of the document with all sections, see nature.com/documents/nr-reporting-summary-flat.pdf

Life sciences study design

All studies must disclose on these points even when the disclosure is negative.

Sample size	Our laboratory has performed many studies with the animal models utilized in this manuscript. Sample sizes were chosen based upon our extensive paast experience using these models for testing other therapeutic agents.
Data exclusions	No data were excluded.
Replication	Multiple time points and multiple doses were tested for sunitinib MPs providing multiple replications of efficacy that were in agreement.
Randomization	One eye was selected for experimental intervention and the other eye of the same animal was used as control. The eyes of each animal behave very similarly and therefore there was no need to alter eye selection among animals which increases chances of mislabeling. There was no need to randomize animals because they were all treated the same.
Blinding	Two investigators worked together. After removal and processing of eyes, retinal flat mounts were coded by one investigator and the other investigator performed measurements with image analysis masked with respect to treatment.

Reporting for specific materials, systems and methods

We require information from authors about some types of materials, experimental systems and methods used in many studies. Here, indicate whether each material, system or method listed is relevant to your study. If you are not sure if a list item applies to your research, read the appropriate section before selecting a response.

Materials & experimental systems

n/a	Involved in the study	
[ ]	[x]	Antibodies
[x]	[ ]	Eukaryotic cell lines
[x]	[ ]	Palaeontology
[ ]	[x]	Animals and other organisms
[x]	[ ]	Human research participants
[x]	[ ]	Clinical data

Methods

n/a	Involved in the study	
[x]	[ ]	ChIP-seq
[x]	[ ]	Flow cytometry
[x]	[ ]	MRI-based neuroimaging

Antibodies

Antibodies used	1) Grk1 antibody (Catalog no. PA5-13725, Lot no. RG22174514, ThermoFischer Scientific Carlsbad, CA), 2) Actin (Catalog no. A2066, Lot no. 019M4777V, Sigma Life Science, St. Louis, MO). 3) Goat anti-rabbit HRP antibody (Catalog no. GENA934, Lot no. 16938431, Millipore Sigma, St Louis, MO).
Validation	1) GRK1 antibody has been validated on mouse lysates in western blots in the manufacturer's website (https://www.thermofisher.com/antibody/product/GRK1-Antibody-Polyclonal/PA5-13725).<br>2) Actin antibody has been validated in western blots on mouse lysates. The validation of the antibody and related citations are in the manufacturer's website (https://www.sigmaaldrich.com/catalog/product/sigma/a2066?lang=en&region=US).<br>3) Goat anti-rabbit HRP antibody validation and related citations are in the manufacturer's website (https://www.sigmaaldrich.com/catalog/product/sigma/gena9341ml?lang=en&region=US).

Animals and other organisms

Policy information about studies involving animals; ARRIVE guidelines recommended for reporting animal research

Laboratory animals	1). For the CNV experiments, wildtype C57BL/6N mice, females of 4 weeks were used (Charles River, Wilmington, MA).<br>2). For quantification of sub-retinal neovascularization and assessments of photoreceptor survival, Rho/VEGF mice in C57BL/6 background, of either sex at P14 were injected but sacrificed at different ages as described in the manuscript.<br>3). For measurement of retinal vascular leakage with Evans Blue dye and leukostasis, C57BL/6N of either sex, at 5-7 weeks were used.

	4) For Pharmacokinetic studies in mice, C57BL/6N mice of either sex at 5-7 weeks were used. For rabbit studies, male pigmented New Zealand rabbits, at 8 months were used.
Wild animals	This study did not involve wild animals.
Field-collected samples	This study did not involve samples collected from the field.
Ethics oversight	Mice were treated in accordance with the Association for Research in Vision and Ophthalmology Guidelines on the care and use of animals in research.

Note that full information on the approval of the study protocol must also be provided in the manuscript.
